# The use of multiple datasets to identify autophagy-related molecular mechanisms in intracerebral hemorrhage

**DOI:** 10.3389/fgene.2023.1032639

**Published:** 2023-04-03

**Authors:** Yinggang Xiao, Yang Zhang, Cunjin Wang, Yali Ge, Ju Gao, Tianfeng Huang

**Affiliations:** ^1^ Department of Anesthesiology, Clinical Medical College of Yangzhou University, Yangzhou, Jiangsu, China; ^2^ Department of Anesthesiology, Yangzhou University Affiliated Northern Jiangsu People’s Hospital, Yangzhou, Jiangsu, China; ^3^ Yangzhou Key Laboratory of Anesthesiology, Yangzhou, Jiangsu, China

**Keywords:** intracerebral hemorrhage, autophagy, immune infiltration, bioinformatics analysis, ceRNA network

## Abstract

**Background:** Intracerebral hemorrhage (ICH) is a stroke syndrome with high mortality and disability rates, but autophagy’s mechanism in ICH is still unclear. We identified key autophagy genes in ICH by bioinformatics methods and explored their mechanisms.

**Methods:** We downloaded ICH patient chip data from the Gene Expression Omnibus (GEO) database. Based on the GENE database, differentially expressed genes (DEGs) for autophagy were identified. We identified key genes through protein–protein interaction (PPI) network analysis and analyzed their associated pathways in Gene Ontology (GO) and the Kyoto Encyclopedia of Genes and Genomes (KEGG). Gene-motif rankings, miRWalk and ENCORI databases were used to analyze the key gene transcription factor (TF) regulatory network and ceRNA network. Finally, relevant target pathways were obtained by gene set enrichment analysis (GSEA).

**Results:** Eleven autophagy-related DEGs in ICH were obtained, and *IL-1B*, *STAT3*, *NLRP3* and *NOD2* were identified as key genes with clinical predictive value by PPI and receiver operating characteristic (ROC) curve analysis. The candidate gene expression level was significantly correlated with the immune infiltration level, and most of the key genes were positively correlated with the immune cell infiltration level. The key genes are mainly related to cytokine and receptor interactions, immune responses and other pathways. The ceRNA network predicted 8,654 interaction pairs (24 miRNAs and 2,952 lncRNAs).

**Conclusion:** We used multiple bioinformatics datasets to identify *IL-1B*, *STAT3*, *NLRP3* and *NOD2* as key genes that contribute to the development of ICH.

## 1 Introduction

Intracerebral hemorrhage (ICH) is a common stroke syndrome, accounting for approximately 15% of strokes, and nearly 50% of stroke-related deaths worldwide are related to ICH (Feigin et al., 2009; Biffi et al., 2016). ICH is caused by the sudden rupture of blood vessels caused by pathological accumulation of blood in the brain parenchyma (Jia et al., 2020). ICH injury is divided into primary and secondary injuries, with the former being caused by direct mechanical action of the hematoma (Fu et al., 2022). Edema around the hematoma occurs within hours of ICH, disrupting the blood–brain barrier and adjacent tissues and leading to secondary damage (Li et al., 2020). Second, mitochondrial dysfunction, neurotransmitter disturbance, microglial activation, and the release of inflammatory mediators are also important mechanisms for aggravating brain injury (Kim-Han et al., 2006). The death of nerve cells after ICH is closely related to the sequelae of ICH and death from ICH. Programmed cell death (PCD) refers to the autonomous and orderly death of cells controlled by genes to maintain the stability of the internal environment. PCD is an active suicidal behavior of cells (Huysmans et al., 2018). PCD, including autophagy, apoptosis and pyroptosis, plays an important role in neuronal cell death after ICH (Bobinger et al., 2018). Autophagy, as an important category of PCD, has been identified in ICH, but its mechanism in intracerebral hemorrhage remains unclear.

Autophagy is one of the important subcellular events occurring from eukaryotic cells to mammals, and the process of autophagy is highly conserved. Autophagy refers to the process in which cells can wrap their intracellular contents under stress and integrate with lysosomes to degrade into these contents into biomacromolecules, which are reused by cells (Ohsumi, 2014). Recent studies have shown that autophagy is closely related to the occurrence of various neurological diseases (Moujalled et al., 2021). In recent years, autophagy has been found to be closely related to secondary brain tissue damage after ICH (Duan et al., 2016; Zhang et al., 2021). After ICH occurs, thrombin is produced in the blood coagulation process, while the hematoma gradually degrades, releasing degradation products such as hemoglobin, heme and iron that invade the surrounding brain tissue. When iron overload and abnormal thrombin expression occur in brain tissue, autophagy is activated and involved in the brain protection process to reduce injury, remove harmful substances and maintain intracellular environmental homeostasis. The protective role of autophagy in ICH has been demonstrated (Wang et al., 2020; Li et al., 2021). However, the overactivation of autophagy, which activates microglia to produce proinflammatory factors and damages neurons, leads to the aggravation of secondary injury after ICH (Shi et al., 2018; Zhang et al., 2021). In summary, autophagy is extremely important for the progression of ICH, but the key genes involved in this process are still not clearly known. The diagnosis of the severity of ICH on the basis of autophagy-related gene expression is also a clinical blind spot. The key autophagy-related genes in ICH need to be identified.

To explore and identify potential biomarkers and the key autophagy-related genes in ICH, we obtained microarray and gene information from multiple databases and used the R statistical programming language for analysis. We selected DEGs in perihematomal tissue (PH) and contralateral normal tissue from intracerebral hemorrhage patients obtained from multiple sources as raw data. Then, four key genes were screened by analyzing the interactions and relationships of DEGs highly related to autophagy with the ROC curve method. Finally, we analyzed the impact of key genes on the immune microenvironment and the mechanisms by which these genes are regulated by transcription factors and non-coding RNAs. We innovatively used methods such as ceRNA network construction, motif-TF annotation and xCell to analyze autophagy after ICH. These results will contribute to the study of the mechanism of secondary injury following ICH and provide new ideas for the diagnosis and treatment of ICH in the clinic.

## 2 Materials and methods

### 2.1 Data download

**FIGURE 1:**
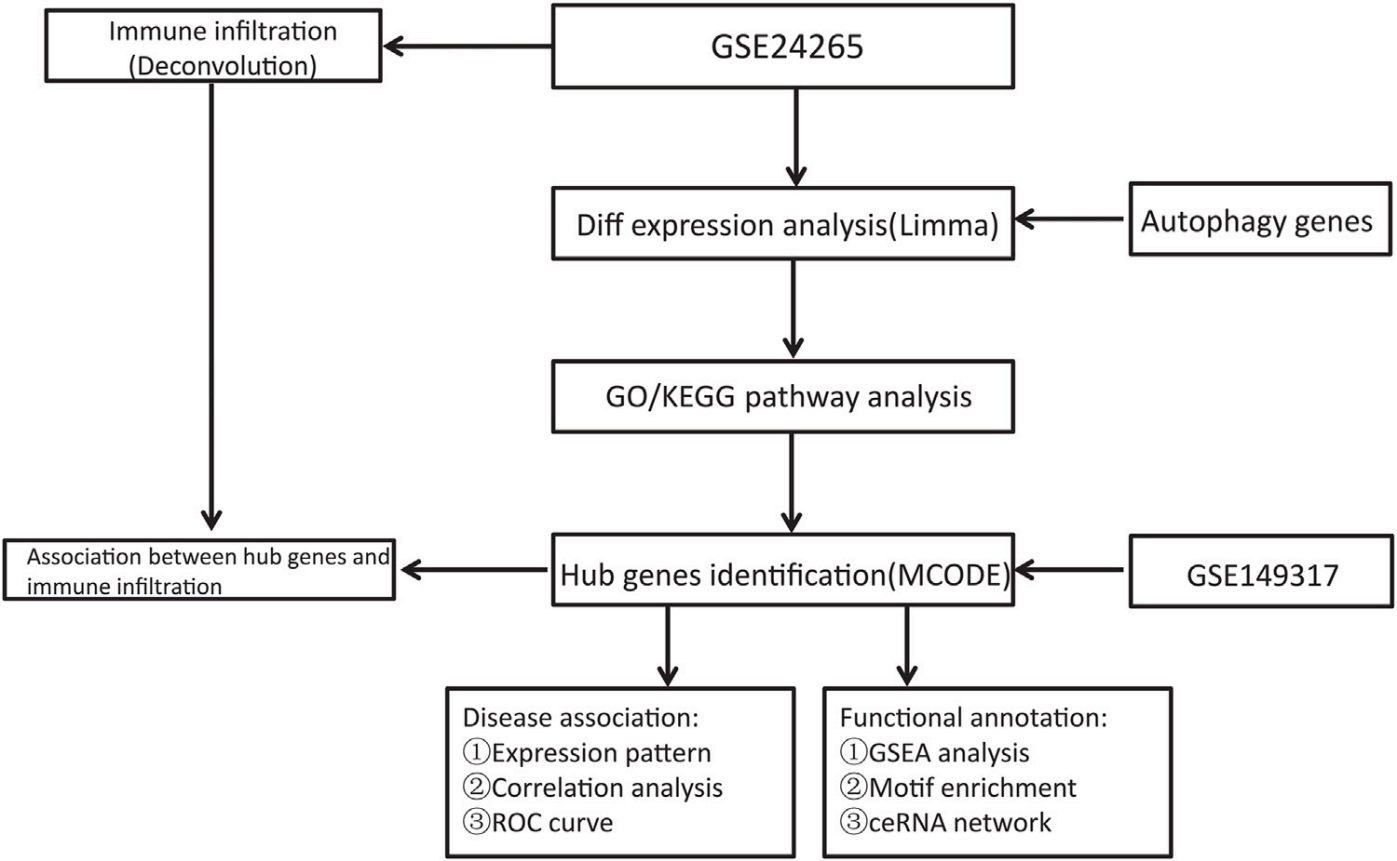
Flow chart.

The NCBI GEO Database (http://www.ncbi.nlm.nih.gov/geo/) is a repository of microarray, next-generation sequencing, and other high-throughput sequencing data (Edgar et al., 2002). The GSE24265 Series Matrix File was downloaded from the GEO public database, noted by the GPL570 annotation file, of which the expression profile data belonged to 11 samples, including the perihematomal areas, gray matters, and white matters of 7 patients in the healthy control group and 4 patients with ICH (Rosell et al., 2011). The GSE149317 Series Matrix File (only used to verify the expression level of key genes) was downloaded from the GEO public database, and the annotated File is GPL24688 (Yuan et al., 2020). The microarray data included 6 cases in the healthy control group and 6 patients in the ICH group. We used the R package limma to count the differentially expressed genes between ICH patient and healthy control samples (Ritchie et al., 2015). The screening conditions for differential genes were *P*.Value < 0.05 and |logFC| > 1. Using the GeneCards database (https://www.genecards.org/) (Stelzer et al., 2016), 7236 autophagy-related genes were obtained. The relevance scores of 269 genes were greater than 3, and these genes were chosen for analysis as an autophagy gene set. Another 1139 ICH-related genes were also obtained from the database. The flow chart of this study is shown in Figure 1.

### 2.2 Functional annotation

The R package clusterProfiler was used to comprehensively explore the functional correlation of these differentially expressed genes (Yu et al., 2012). GO and KEGG were used for the evaluation of relevant functional categories. GO and KEGG enriched pathways with both *p* values and *q-*values less than 0.05 were considered significant pathways. To comprehensively explore the functional correlation of differentially expressed genes, we also used the Metascape database (www.metascape.org) for gene annotation (Zhou et al., 2019). GO and KEGG were used to analyze the potential pathways of the selected genes. Min overlap ≥ 3 and *p* ≤ 0.01 were considered statistically significant.

### 2.3 Protein–protein interaction network analysis

The protein–protein interaction (PPI) information of genes was retrieved through the STRING database (Szklarczyk et al., 2021), and the confidence scores were set to ≥0.4. Cytoscape software was used to visualize the results, and the gene coexpression network was obtained. The MCODE algorithm of Cytoscape identified densely connected sets of genes in the PPI network.

### 2.4 Analysis of immune cell infiltration

Developed by the Dviraran team in 2017, xCell is a widely used method to evaluate immune cell types in the microenvironment (Aran et al., 2017). This method integrates the strengths of gene enrichment analysis *via* deconvolution to assess 64 cell types that include multiple adaptive and innate immune cells, hematopoietic progenitor cells, epithelial cells, and extracellular stromal cells, including 48 tumor microenvironment-related cells. With the R package xCell, we analyzed the patient data to infer the relative proportion of infiltrating immune cells and performed Pearson correlation analysis on the level of immune cell infiltration. Pearson correlation analysis was used to evaluate the immune cell content and the expression level of some key genes.

### 2.5 Transcription factor regulatory network analysis of key genes

The transcription initiation process of eukaryotes is very complex and often requires the assistance of various protein factors. TFs and RNA polymerase II form a transcription initiation complex and participate in the process of transcription initiation together. TFs can be divided into two categories according to their function. The first category is universal transcription factors, which, when acting together with RNA polymerase II to form the transcription initiation complex, can start transcription at the correct position. Another category is cis-acting elements, which are sequences present in sequences flanking genes that can affect gene expression. Cis-acting elements include promoters, enhancers, regulatory sequences, and inducible elements that participate in the regulation of gene expression. The cis-acting element itself does not encode any protein but provides an action site to interact with the trans-acting factor. This analysis was mainly performed using the R package cisTarget (https://resources.aertslab.org/cistarget/), in which we used mm9-500bp-upstream-7species.mc9nr.feather version 1.6.0 for the Gene-motif rankings database. The main TFs were predicted by the cisTarget function, when nesThreshold was 3, geneErnMethod was aprox, and geneErnMmaxRank was 5000.

### 2.6 Gene set enrichment analysis

According to a predefined set of genes, GSEA is a statistical procedure to rank genes according to their degree of differential expression in two types of samples and then test whether the predefined gene set is enriched at the top or bottom of the ranking list (Subramanian et al., 2005). In this study, GSEA was used to compare the discrepancies in signaling pathways between the high expression group and the low expression group and to explore the molecular mechanisms of the core genes of patients. The number of substitutions was 1000, and the substitution type was phenotype.

### 2.7 Analysis of the ceRNA network

Representing a new mode of gene expression regulation, ceRNA has attracted much attention in the academic community in recent years. Compared with the miRNA regulatory network, the ceRNA network is more elaborate and complex, involving more RNA molecules, including mRNAs, gene-coding pseudogenes, long non-coding RNAs, and miRNAs. In addition, we combined four databases, miRWalk, miRDB, TargetScan and ENCORI, to predict the interaction between key mRNAs and non-coding RNAs. Moreover, we selected coidentified targeted mRNAs for further analysis. Finally, ceRNA networks were established with the combination of mRNA–miRNA and miRNA–lncRNA interactions and visualized with Cytoscape.

## 3 Results

### 3.1 Identification of Hub genes

**FIGURE 2:**
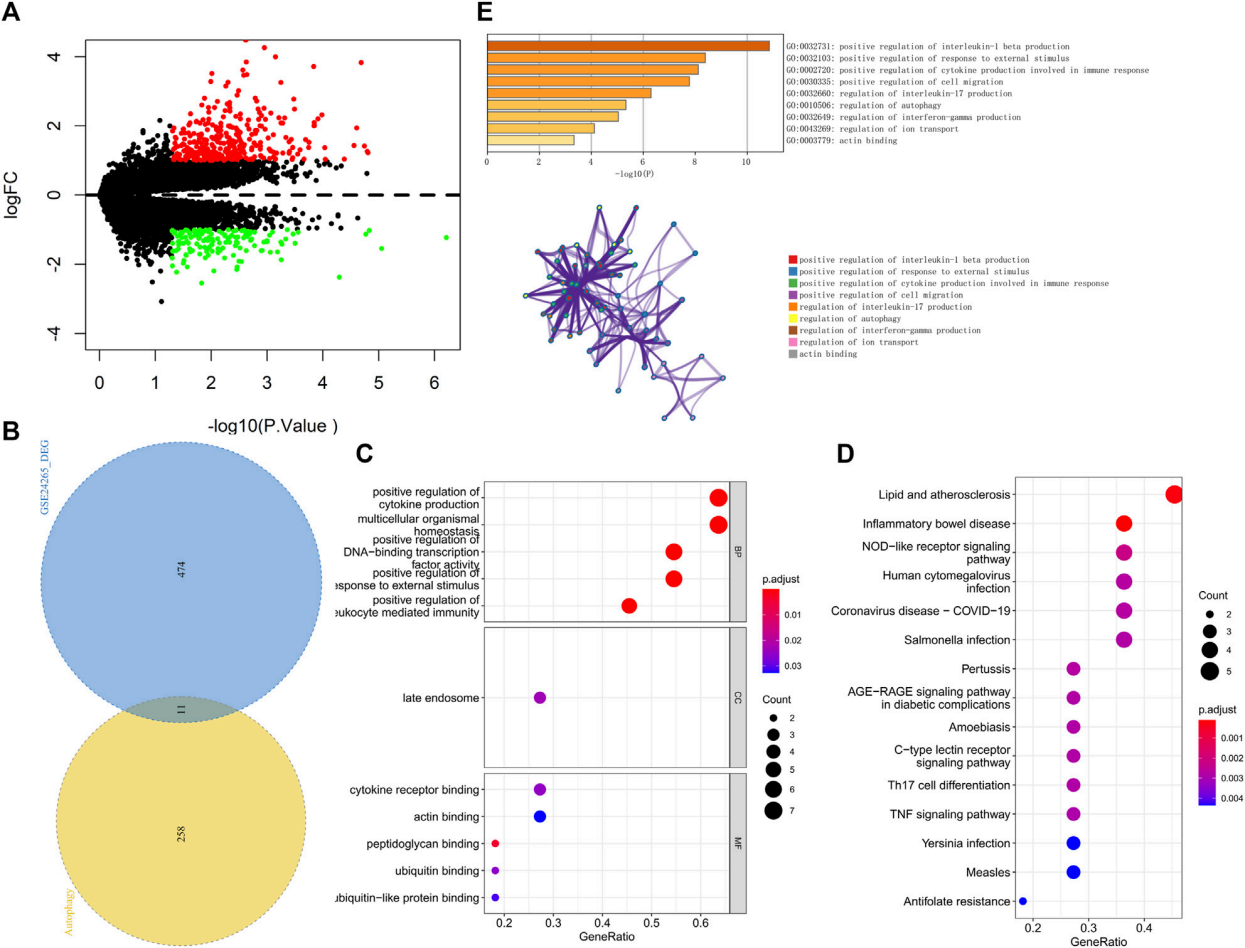
**(A)** The volcano plot of differentially expressed genes in the GSE24265 dataset. Red indicates the downregulation of differentially expressed genes, green indicates the upregulation of differentially expressed genes, and the screening conditions for differentially expressed genes were *P*.Value < 0.05 and |logFC| > 1. **(B)** Venn diagram of differentially expressed genes and autophagy-related genes. **(C)** GO enrichment results of differentially expressed genes, including BP, CC, and MF, sorted according to the number of genes enriched in the pathway. **(D)** KEGG enrichment results of differentially expressed genes sorted according to the number of genes enriched in the pathway. **(E)** GO-KEGG enrichment analysis of differential genes based on the Metascape database are shown above. The cluster networks composed of enriched pathways are shown below, where nodes sharing the same cluster are usually close to each other.

We downloaded the GSE24265 dataset from the NCBI GEO public database, which contained the data from a total of 11 individuals, including 7 in the healthy control group and 4 in the disease group. Through comparison with the healthy control group, we used the limma package to screen out a total of 341 upregulated genes and 144 downregulated genes in the patient samples (Figure 2A). Among them, 11 autophagy-related genes (all upregulated genes) were included (Figure 2B). Ultimately, we used these 11 autophagy-related differentially expressed genes as candidate gene sets for further analysis.

### 3.2 Functional enrichment analysis

We further performed pathway analysis on these 11 candidate genes. GO enrichment analysis showed that these candidate genes were mainly enriched in the positive regulation of cytokine production and cytokine receptor binding pathways (Figure 2C). KEGG enrichment analysis revealed that these candidate genes were mainly enriched in pathways such as lipid and atherosclerosis and the nucleotide-binding oligomerization domain (NOD)−like receptor signaling pathway (Figure 2D). The Metascape database was used for further pathway analysis of candidate genes. The results showed that these candidate genes were mainly enriched in positive regulation of interleukin-1 beta production, the regulation of interleukin-17 production and the regulation of autophagy pathways (Figure 2E).

### 3.3 Identification of key genes and ROC curve analysis

**FIGURE 3:**
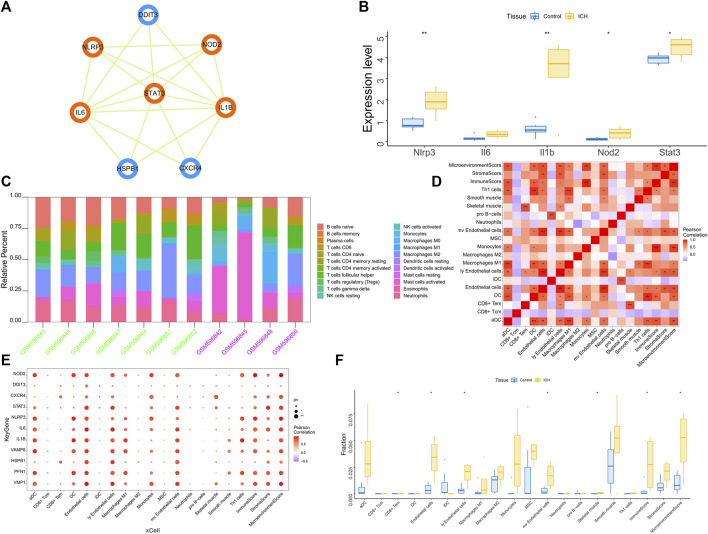
**(A)** Based on the Cytoscape software, the key clusters obtained by the protein interaction network MCODE algorithm, the orange genes are the key genes in the cluster. **(B)** The GSE149317 dataset validates the 5-based expression, with blue for the control group and yellow for the ICH group. The test method was ANOVA. **(C)** Relative percentages of 20 immune cell subsets per sample. On the horizontal axis, green is the healthy control group, and purple is the patient group. **(D)** Pearson correlation between 20 immune cells; purple indicates a negative correlation, and red indicates a positive correlation. The *p* value of the level of correlation is indicated by an asterisk: * for *p* < 0.05, ** for *p* < 0.01, and *** for *p* < 0.001. **(E)** Pearson correlation analysis of 11 candidate genes and 20 types of immune cells; purple indicates a negative correlation, and red indicates a positive correlation. **(F)** The difference in immune cell content between healthy controls and ICH patients (yellow indicates healthy controls, and blue indicates ICH patients); *p* < 0.05 was considered statistically significant.

We found multiple protein interaction pairs among 11 candidate genes through the STRING online database. Moreover, five key genes, including *IL1B*, *STAT3*, *IL6*, *NOD2* and *NLRP3*, were obtained by MCODE analysis in Cytoscape (Figure 3A). Then, we analyzed the expression levels of these five key genes in the GSE149317 dataset and found that the expression levels of interleukin-1beta (*IL1B*), signal transducer and activator of transcription 3 (*STAT3*), nucleotide-binding oligomerization domain containing 2 (*NOD2*) and NOD-1-like receptor pyrin domain containing three (*NLRP3*) were significantly higher in the ICH group than in the healthy control group (Figure 3B). The area under the receiver operating characteristic curve (AUC) for the four key genes was no less than 0.75 (Supplementary Figure S1). Based on the GSE24265 dataset, we once again analyzed the predictive power of these key genes for ICH. The results showed that the AUCs of *IL1B*, *STAT3*, *NOD2* and *NLRP3* were greater than 0.8 (Supplementary Figure S1).

### 3.4 Analyses of the immune microenvironment

The immune microenvironment is mainly composed of immune-related fibroblasts, immune cells, extracellular matrix, various growth factors, inflammatory factors and special physicochemical characteristics. The immune microenvironment significantly affects the diagnosis, survival outcome and clinical severity of disease. Analyzing the relationship between core genes and immune infiltration in the GSE24265 dataset, we further explored the potential molecular mechanisms affecting disease progression. The 20 most significant immune factors in the Wilcoxon test were selected for analysis. The research results showing the proportion of immune cells and the correlation with immunity are shown in Figures 3C, D. There were multiple significant correlation pairs between the expression level of candidate genes and the level of immune infiltration (Figure 3E). In addition, the levels of endothelial cells and Ly endothelial cells in the ICH group were higher than those in the healthy controls (Figure 3F). We further explored the relationship between key genes and immune cells and found that key genes were mostly positively correlated with immune cell infiltration levels. For example, Endothelial cells, MicroenvironmentScore, aDC, ly Endothelial cells and ImmuneScore were significantly positively correlated with 4 key genes, but CD8^+^ Tcm, iDC and pro B−cells were significantly negatively correlated with key genes (Supplementary Figure S2). We further obtained the correlations between these key genes and different immune factors from the TISIDB database, including immunomodulators, chemokines and cell receptors (Supplementary Figure S3). These data confirmed that these key genes are closely related to immune cell infiltration levels and play important roles in the immune microenvironment.

### 3.5 The correlation between key genes and ICH-related genes

We obtained 1,139 ICH-related pathogenic genes through the GeneCards database. Based on the GSE24265 dataset, we analyzed the expression levels of the 4 key genes and the top 20 genes in the Relevance score from GeneCards. Statistical analysis by ANOVA showed that the expression levels of these disease-related genes were significantly different between the healthy control group and the disease-related group. In addition, the expression levels of key genes were significantly correlated with the expression levels of multiple disease-related genes (Supplementary Figure S4).

### 3.6 Transcription factors of key genes

**FIGURE 4:**
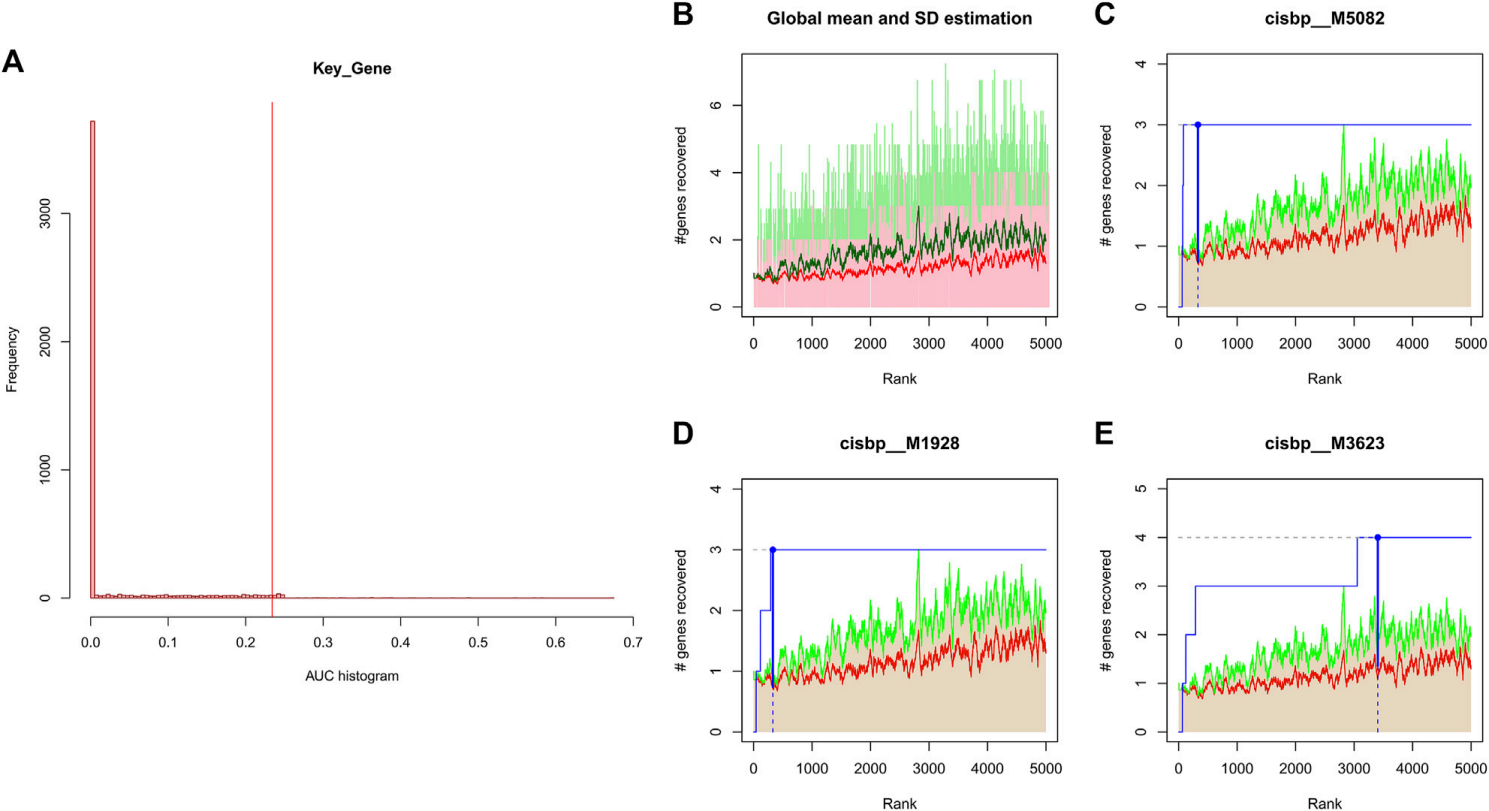
**(A)** Distribution of AUC values for enriched motifs, which were calculated from the recovery curves of key genes for motif ordering. **(B–E)** In the figure, the red line is the mean value of the recovery curve of each motif, the green line is the mean + standard deviation, and the blue line is the recovery curve of the current motif.

We applied these four key genes to the gene set for this analysis and found that they are regulated by a common mechanism including multiple transcription factors. Therefore, enrichment analysis (Figure 4), motif-TF annotation and the selection of important genes were performed for these transcription factors using accumulative recovery curves. The analysis results showed that the motif with the highest normalized enrichment score (NES: 7.70) was annotated as cisbp__M5082. Three genes were enriched in this motif, namely, *IL1B*, *NLRP3* and *NOD2*. We displayed all enriched motifs and corresponding transcription factors of core genes (Supplementary File S1)

### 3.7 GSEA of key genes

**FIGURE 5:**
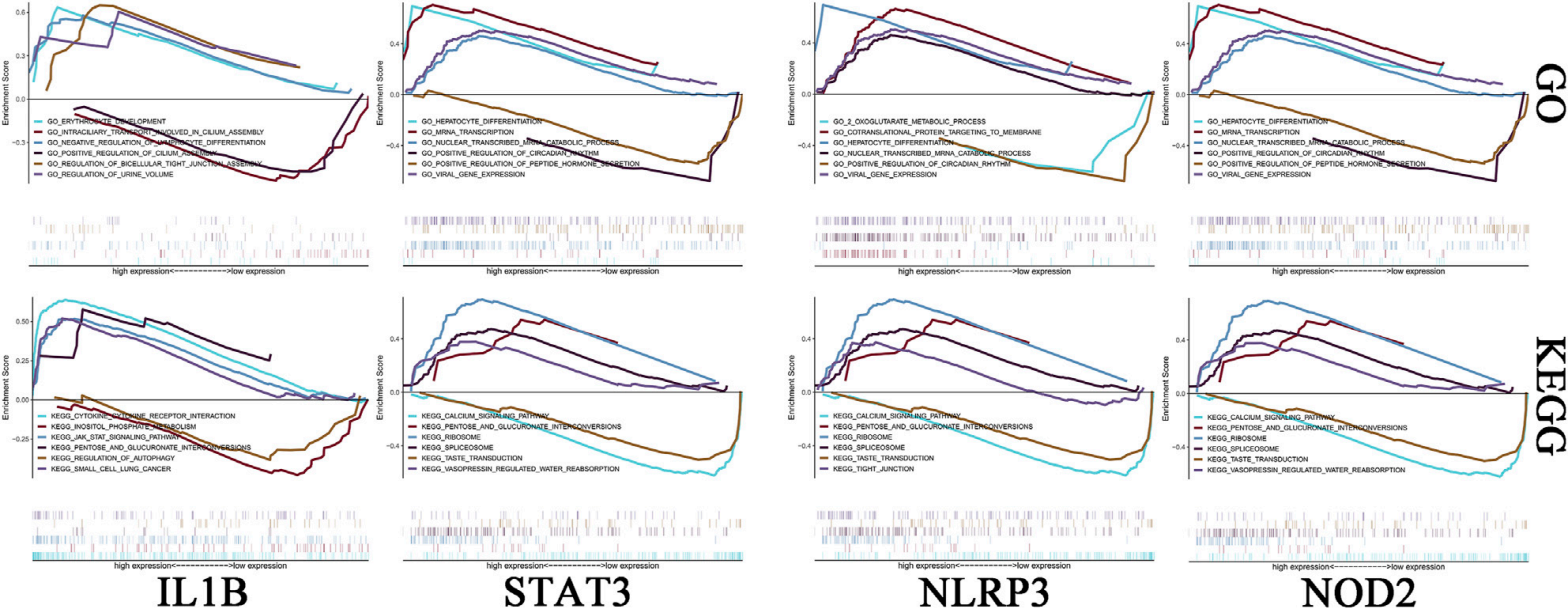
Enrichment analysis of key genes. The first row is the GO analysis, and the second row is the KEGG analysis. From left to right are *IL-1B*, *STAT3*, *NLRP3* and *NOD2*.

We investigated the specific signaling pathways enriched by the 4 key genes and explored the underlying molecular mechanisms by which the core genes affect the progression of ICH. Some of these highly significant pathways were selected to be displayed in detail (Figure 5). The *IL1B* gene GO enrichment pathways were ERYTHROCYTE DEVELOPMENT, INTRACILIARY TRANSPORT INVOLVED IN CILIUM ASSEMBLY, etc. The *IL1B* gene KEGG enrichment pathways were CYTOKINE-CYTOKINE RECEPTOR INTERACTION, JAK STAT SIGNALING PATHWAY, etc. The *NLRP3* gene GO enrichment pathways were COTRANSLATIONAL PROTEIN TARGETING TO MEMBRANE, 2 OXOGLUTARATE METABOLIC PROCESS, etc. The *NLRP3* gene KEGG enrichment pathways were CALCIUM SIGNALING PATHWAY, PENTOSE AND GLUCURONATE INTERCONVERSIONS. The *NOD2* gene GO enrichment pathways were CELLULAR METABOLIC COMPOUND SALVAGE, HISTONE H4 K16 ACETYLATION, etc. The *NOD2* gene KEGG enrichment pathways were ANTIGEN PROCESSING AND PRESENTATION, AUTOIMMUNE THYROID DISEASE, etc. The *STAT3* gene GO enrichment pathways were HEPATOCYTE DIFFERENTIATION, MRNA TRANSCRIPTION, etc. The *STAT3* gene KEGG enrichment pathways were CALCIUM SIGNALING PATHWAY, PENTOSE AND GLUCURONATE INTERCONVERSIONS, etc.

### 3.8 Further ceRNA interaction and mining

**FIGURE 6:**
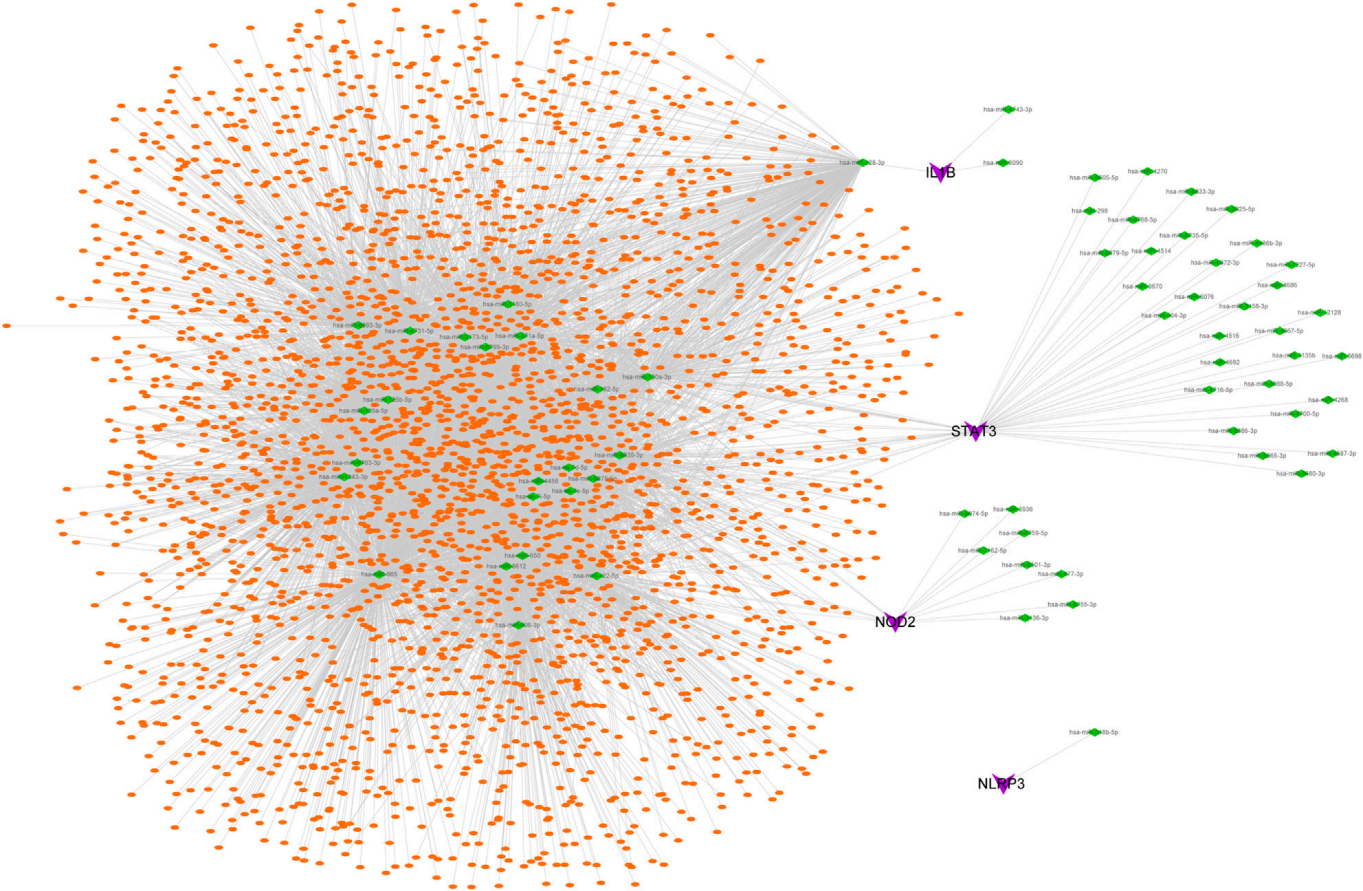
CeRNA network of key genes. Purple represents key genes, green represents miRNA, and orange represents lncRNA.

The possible miRNAs and lncRNAs of the 4 key genes were obtained from the miRWalk database and ENCORI database, respectively. First, the four key mRNA-related mRNA–miRNA relationship pairs were extracted from the miRWalk database, but we retained only 67 mRNA–miRNA pairs (4 mRNAs and 66 miRNAs) that were validated in TargetScan or miRDB. Then, interacting lncRNAs were predicted based on these miRNAs, and a total of 8,654 pairs of interactions (24 miRNAs and 2,952 lncRNAs) were predicted. Finally, a ceRNA network was constructed by Cytoscape (V3.7) (Figure 6).

## 4 Discussion

Defined as a primary, non-traumatic intraparenchymal hemorrhage, ICH can lead to severe disability and is associated with a high fatality rate of 30%–50% within 6 months (Mayer and Rincon, 2005). The mortality rate of ICH within 30 days is 32%–50%, and only 28%–35% of patients who survive 3 months are able to live independently (Martini et al., 2012). As a subtype of stroke, the pathogenesis and treatment of ICH have been extensively studied, and there is still a lack of effective acute treatment. Autophagy, as an important regulatory mechanism of intracellular homeostasis, has been gradually recognized in ICH, but the regulatory effects of autophagy on intracellular homeostasis and the immune microenvironment after ICH remain to be further explored.

Inflammation in secondary injury after ICH is mainly due to the activation of and increase in inflammatory cells and inflammatory factors (Wang, 2010). After ICH, components in the blood, including blood cells, cytokines and various immune cells, quickly cross the blood–brain barrier and enter and accumulate at the center of the injured site. This is followed by the activation of infiltrated immune cells and immune cells of the central nervous system, including the polarization of macrophages and microglia, the activation of leukocytes and astrocytes, and brain tissue damage and repair (Xue and Del Bigio, 2000; Sheth and Rosand, 2014). Immune cells include peripheral blood-derived leukocytes and macrophages, innate microglia, astrocytes, and mast cells. Many studies have shown that leukocytes, macrophages, activated microglia, and astrocytes are the main cellular mediators of secondary injury in ICH (Illanes et al., 2011). These immune cells can release cytokines, chemokines, prostaglandins, proteases, ferrous iron, and other immunologically active molecules (Hua et al., 2006). The R package X cell analysis indicated that four key genes can cause macrophages, neutrophils and CD8^+^ T cells to infiltrate the lesions during ICH and can also promote the increase in related immune factors and aggravate the inflammatory response.

Although the pathogenesis of ICH has been extensively studied at the transcriptional level, there are some limitations of these studies. Most of the related research at the RNA level is on only the regulatory relationship between a single type of RNA, such as lncRNAs, miRNAs or mRNAs, and ICH, but little is known about the interaction of different RNAs in the development of ICH. The discovery of ceRNAs in recent years has solved this problem. ceRNA refers to RNA that has a miRNA binding site and can compete with mRNA to bind miRNA, thereby inhibiting the regulatory effect of miRNA on target genes (Ma et al., 2020). The ceRNA regulatory network rigorously integrates the mutual regulatory relationship between mRNA and non-coding RNA (ncRNA), providing significant help for the study of posttranscriptional mechanisms of diseases (Qi et al., 2015). Numerous studies have shown that the ceRNA regulatory network plays an important role in secondary injury following ICH (Liu et al., 2021; Wang et al., 2021; Yang et al., 2022). Based on multiple databases, the key gene-related ceRNA network described in this study shows miRNAs and related lncRNAs that play major regulatory roles.

This study identified *IL-1B*, *STAT3*, *NOD2* and *NLRP3* as key causative genes for secondary injury in ICH and demonstrated the critical role of autophagy in ICH. We combined two datasets, mainly using GO/KEGG analysis, immune infiltration analysis and ceRNA network construction, to screen key autophagy-related genes and analyze their mechanisms affecting ICH progression.

Recent studies have shown that cytokines, including proinflammatory cytokines and anti-inflammatory cytokines, play an important regulatory role in the course of various inflammatory-related diseases. *IL-1B*, a member of the interleukin 1 cytokine family, is a key proinflammatory factor that plays an important role in the body’s immune response and regulates inflammatory responses to brain injury (You et al., 2020). After inflammation occurs in the body, the secretion of IL-1B increases rapidly. In general, IL-1B has a proinflammatory effect in the local inflammatory response, causing vascular dilation and inducing the transfer of monocytes and neutrophils to the inflammatory site, resulting in a stress response and tissue damage (Schett et al., 2016). Our GSEA suggested that *IL-1B* was involved in the process of cytokine binding to its corresponding receptor, which also suggested that *IL-1B* plays an important role in the inflammatory response to ICH. In addition, KEGG analysis of *IL-1B* also showed enrichment of the Janus kinase-signal transducer and activator of transcription (*JAK-STAT*) pathway. Previously, researchers found that miRNAs/mRNAs changes in whole-blood samples for patients with ICH were important links with the *JAK-STAT* pathway (Cheng et al., 2020). The *JAK-STAT* pathway has also been associated with ICH progression in rat models (Ji et al., 2020). Our GSEA results also showed that *STAT3*, which is closely related to mRNA catabolism, is a key gene leading to ICH. The STAT protein family, which includes seven members, plays a key role in regulating cytokine-dependent inflammation and immunity. *STAT3* is considered to be the most conserved and can be activated by various factors and stimuli, such as cytokines and chemokines. *STAT3* is closely related to ischemic stroke and ischemia–reperfusion injury, and its high expression aggravates nerve damage (Zhu et al., 2021). Zhu H reported that *STAT3* activation can promote the occurrence and development of inflammation, leading to increased cerebral edema after ICH and damage to neurons around the hematoma, and *NLRP3* is a downstream molecule of *STAT* (Lee et al., 2006). In addition, the findings from mouse experiments suggest that *NLRP3* is the key to the aggravation of ICH injury caused by *STAT3* (Ji et al., 2022). Our results showed that *NLRP3* was significantly upregulated in the brain tissues of ICH patients, and the AUC of *NLRP3* was greater than 0.89, which indicates that *NLRP3* is a key gene for ICH and has strong predictive value for ICH. NLRP3, a member of the intracytoplasmic pattern recognition receptor NOD-like receptors (NLRs), is an important part of the innate immune system and plays an important regulatory role in the process of innate immune inflammation. *NLRP3* can sense tissue cell damage and is then activated by a variety of damage-associated molecular patterns (DAMPs) or pathogen-associated molecular patterns (PAMPs) (Mangan et al., 2018). Activated NLRP3 protein can form the NLRP3 inflammasome, which can cleave biologically inactive pro-IL-1B into IL-1B and exert its proinflammatory effect (Mangan et al., 2018). The last key gene identified in our analysis, *NOD2*, is also one of the main NLRs. As an important intracytoplasmic pattern recognition receptor, NOD2 is widely involved in the recognition of immune cells and the induction of inflammatory responses (Huang et al., 2013). Activated NOD2 receptors recruit the downstream signaling molecule receptor interacting protein 2 (RIP2), which can activate the non-canonical transcription factor nuclear factor-kappaB (*NF-κB*) and then transcribe NF-κB-dependent target genes, secreting inflammatory factors such as tumor necrosis factor-A (TNF-A) and IL-1B. Although many *NOD2* studies have focused on inflammatory bowel disease, it has been shown that *NOD2* is involved in the inflammatory response after cerebral ischemia, triggering an excessive inflammatory response and exacerbating brain injury (Kuban et al., 2017). This study is the first to suggest that *NOD2* may be a key gene in the development of ICH. Our GSEA results suggest a high correlation of *NOD2* with ANTIGEN PROCESSING AND PRESENTATION.

## 5 Conclusion

In this study, the existing ICH patient data in the GEO database were analyzed by combining autophagy-related genes in the GENE database, and 11 potential pathogenic genes were finally obtained. Finally, with diagnostic and predictive value, *IL-1B*, *STAT3*, *NLRP3* and *NOD2* were obtained through PPI analysis and ROC curve analysis. Then, based on the database and R package, we found that these 4 key genes cause immune cell infiltration into ICH lesions. GSEA revealed the specific signaling pathways involved in key genes, and we explored the possibility that these pathways might influence the development of ICH. The demonstration of TFs and ceRNA networks affecting key genes provides a theoretical basis for TFs and ncRNA in the regulation of the expression of these key genes. The identification of four key genes contributes to the understanding of the mechanism of ICH and provides potential targets and directions for the clinical treatment of ICH.

## Data Availability

The datasets presented in this study can be found in online repositories. The names of the repository/repositories and accession number(s) can be found in the article/Supplementary Material.

## Supplementary material

SUPPLEMENTARY FIGURE S1 The ROC curves of key genes suggest that the genes have good predictive performance for ICH. The first row is the GSE149317 dataset, and the second row is the GSE24265 dataset.

SUPPLEMENTARY FIGURE S2
**(A-D)** Pearson correlation between key genes and immune cells. IL-1B, STAT3, NLRP3 and NOD2 are listed in alphabetical order. **(E)** Pearson correlation analysis of 4 key genes and 20 kinds of immune cells; purple indicates a negative correlation, and red indicates a positive correlation.

SUPPLEMENTARY FIGURE S3
**(A-D)** Pearson correlations of key genes and various immune factors; purple indicates a negative correlation, and red indicates a positive correlation. Chemokine-related genes, receptor-related genes, immunoinhibitor-related genes, MHC-related genes and immunostimulator-related genes are listed in alphabetical order. The *p* value of the level of correlation is indicated by an asterisk: * for *p* < 0.05, ** for *p* < 0.01, and *** for *p* < 0.001.

SUPPLEMENTARY FIGURE S4
**(A)** Differences in the expression of ICH disease-regulating genes; blue indicates healthy controls, and yellow indicates ICH patients. **(B)** The middle panel shows the Pearson correlation analysis of ICH disease-regulating genes and key genes. Blue indicates a negative correlation, and red indicates a positive correlation.

SUPPLEMENTARY FILE S1 All the enriched motifs and corresponding transcription factors of core genes are displayed in the document.
