# Supplementary figures and images for: The use of multiple datasets to identify autophagy-related molecular mechanisms in intracerebral hemorrhage
Source: Front Genet. 2023 Apr 3;14:1032639. doi: 10.3389/fgene.2023.1032639 (PMC10106621; doi:10.3389/fgene.2023.1032639)

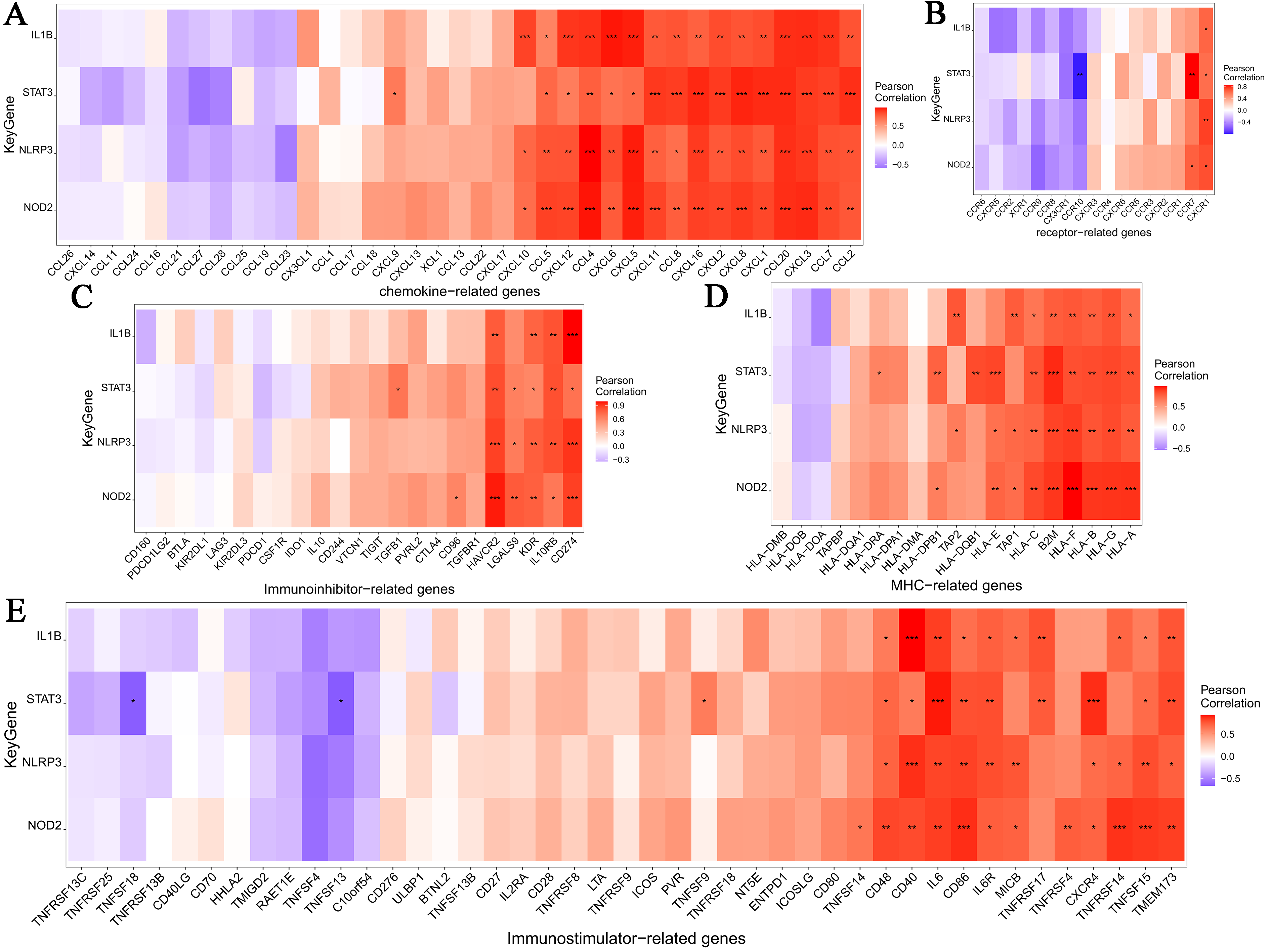

Supplement: Supplementary file 2 [file Image3.TIF]

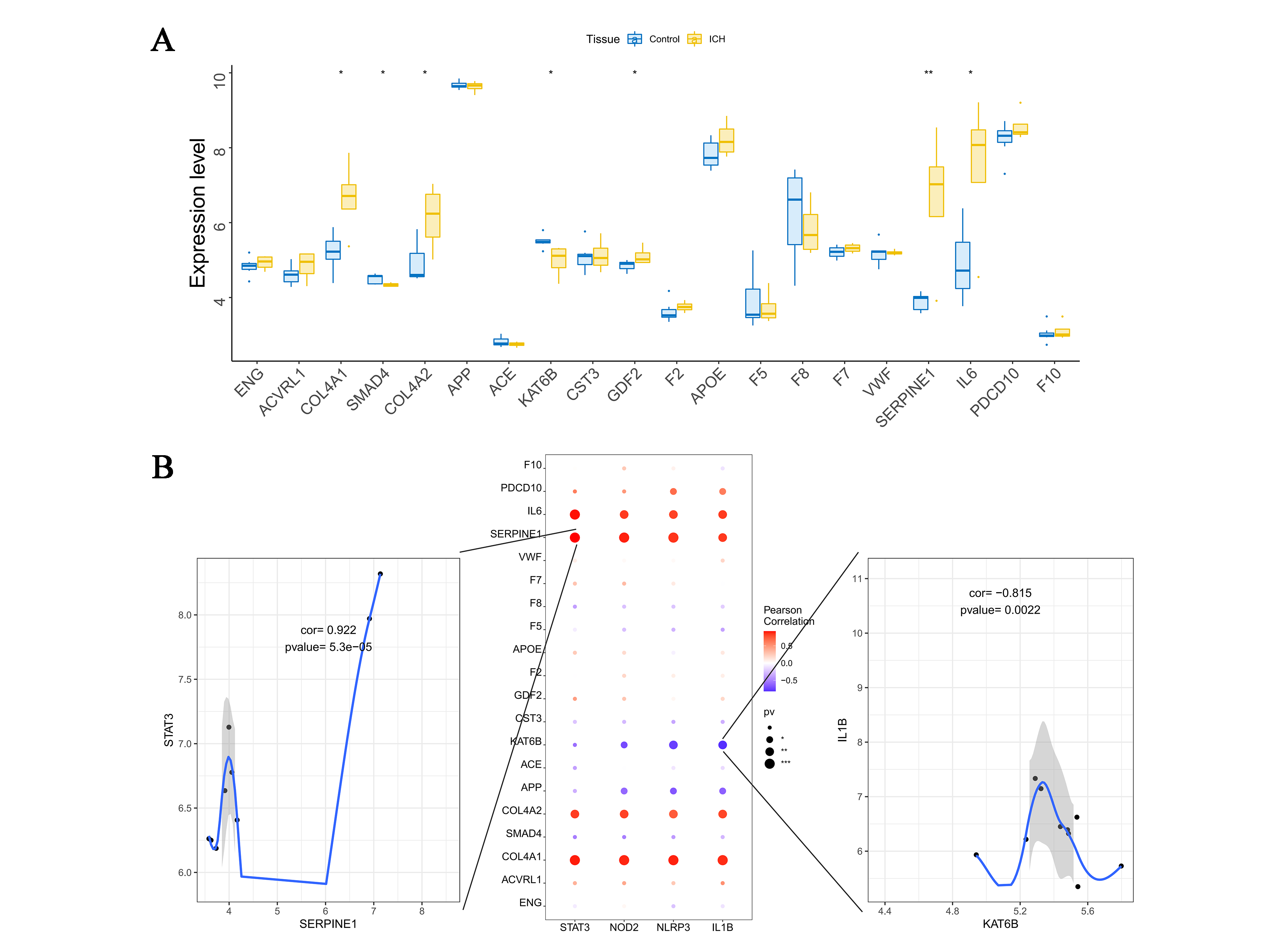

Supplement: Supplementary file 3 [file Image4.TIF]

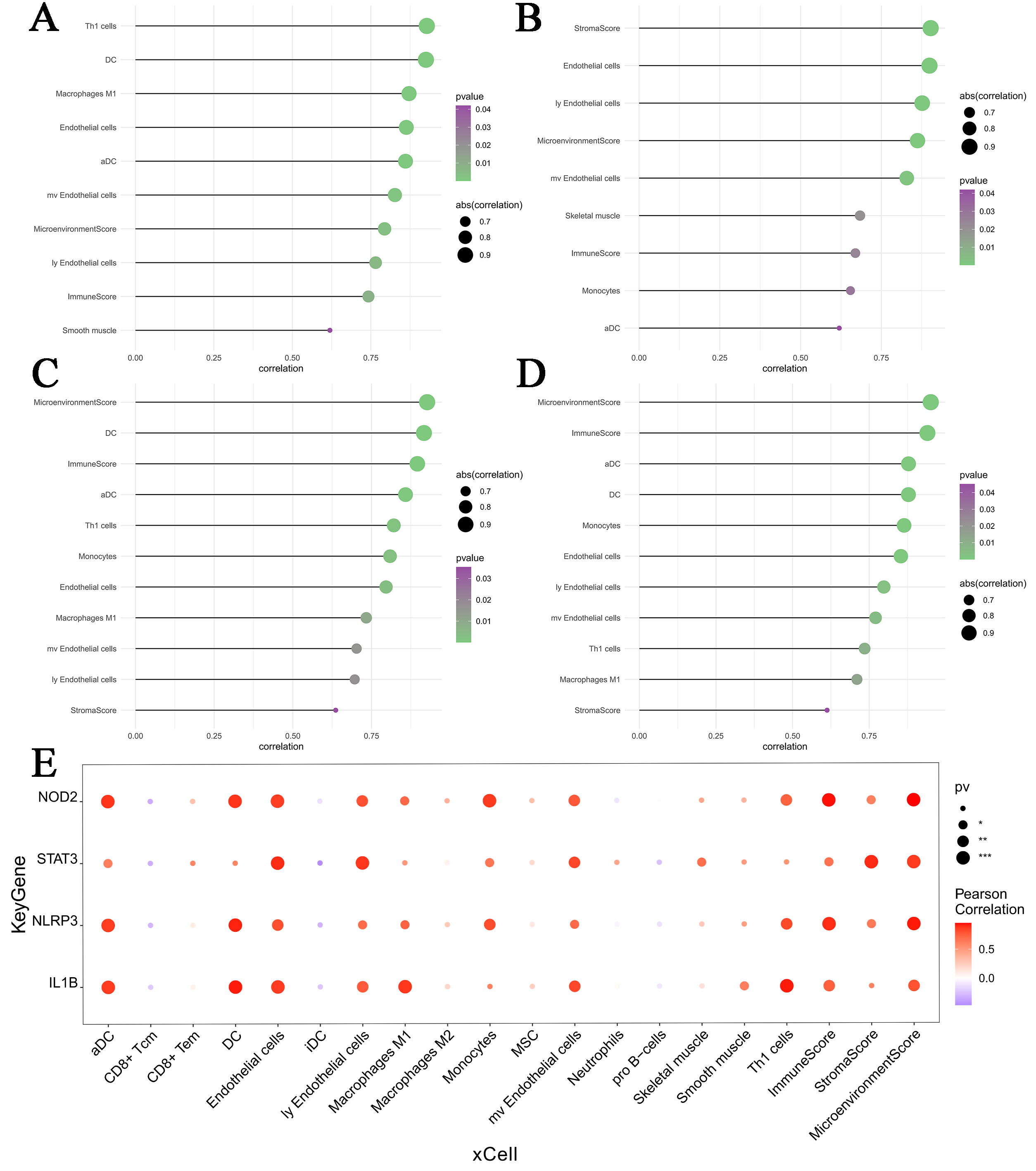

Supplement: Supplementary file 4 [file Image2.TIF]

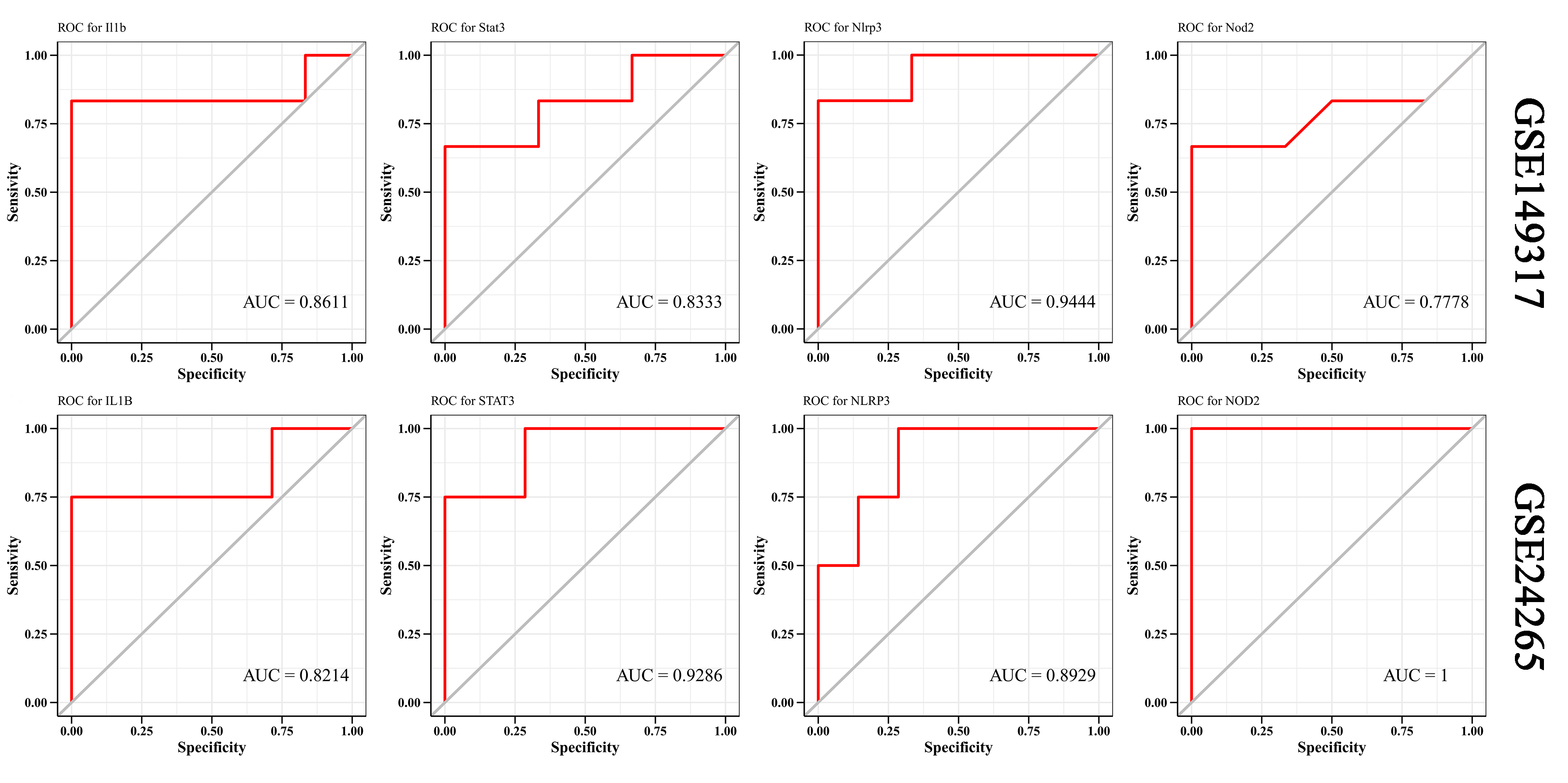

Supplement: Supplementary file 5 [file Image1.TIF]
